# *IL21R* and *PTH* May Underlie Variation of Femoral Neck Bone Mineral Density as Revealed by a Genome-wide Association Study

**DOI:** 10.1359/jbmr.091040

**Published:** 2009-10-26

**Authors:** Yan Guo, Li-Shu Zhang, Tie-Lin Yang, Qing Tian, Dong-Hai Xiong, Yu-Fang Pei, Hong-Wen Deng

**Affiliations:** 1 Key Laboratory of Biomedical Information Engineering of Ministry of Education, and Institute of Molecular Genetics, School of Life Science and Technology, Xi'an Jiaotong University Xi'an, People's Republic of China; 2 School of Medicine, University of Missouri–Kansas City Kansas City, MO, USA; 3 College of Life Sciences and Engineering, Beijing Jiao Tong University Beijing, People's Republic of China; 4 Center of Systematic Biomedical Research, Shanghai University of Science and Technology Shanghai, People's Republic of China

**Keywords:** genome-wide association, BMD, *PTH*, *IL21R*, osteoporosis

## Abstract

Bone mineral density (BMD) measured at the femoral neck (FN) is the most important risk phenotype for osteoporosis and has been used as a reference standard for describing osteoporosis. The specific genes influencing FN BMD remain largely unknown. To identify such genes, we first performed a genome-wide association (GWA) analysis for FN BMD in a discovery sample consisting of 983 unrelated white subjects. We then tested the top significant single-nucleotide polymorphisms (SNPs; 175 SNPs with *p* < 5 × 10^−4^) for replication in a family-based sample of 2557 white subjects. Combing results from these two samples, we found that two genes, parathyroid hormone (*PTH*) and interleukin 21 receptor (*IL21R*), achieved consistent association results in both the discovery and replication samples. The *PTH* gene SNPs, rs9630182, rs2036417, and rs7125774, achieved *p* values of 1.10 × 10^−4^, 3.24 × 10^−4^, and 3.06 × 10^−4^, respectively, in the discovery sample; *p* values of 6.50 × 10^−4^, 5.08 × 10^−3^, and 5.68 × 10^−3^, respectively, in the replication sample; and combined *p* values of 3.98 × 10^−7^, 9.52 × 10^−6^, and 1.05 × 10^−5^, respectively, in the total sample. The *IL21R* gene SNPs, rs8057551, rs8061992, and rs7199138, achieved *p* values of 1.51 × 10^−4^, 1.53 × 10^−4^, and 3.88 × 10^−4^, respectively, in the discovery sample; *p* values of 2.36 × 10^−3^, 6.74 × 10^−3^, and 6.41 × 10^−3^, respectively, in the replication sample; and combined *p* values of 2.31 × 10^−6^, 8.62 × 10^−6^, and 1.41 × 10^−5^, respectively, in the total sample. The effect size of each SNP was approximately 0.11 SD estimated in the discovery sample. *PTH* and *IL21R* both have potential biologic functions important to bone metabolism. Overall, our findings provide some new clues to the understanding of the genetic architecture of osteoporosis. © 2010 American Society for Bone and Mineral Research.

## Introduction

Osteoporosis is a serious public health problem associated with substantive morbidity and mortality,(1) as well as tremendous health care expenditures.(2) It is a common disease characterized by low bone mass and increased risk of fragility fractures. Clinically, bone mineral density (BMD) is the single best predictor of osteoporotic fractures.(3,4) Since hip fracture is the most common and severe form of osteoporotic fractures, and since the risk of hip fracture increases 2.6-fold for each standard deviation (SD) decrease in BMD measured at the femoral neck (FN), low FN BMD is the most important risk factor for osteoporosis at the hip and has been used widely as a reference standard for the description of osteoporosis.(5)

FN BMD is a highly heritable quantitative trait, with estimated heritability over 75%.(6,7) Numerous association or linkage analyses have been conducted to identify candidate genes for BMD, although only a few genes were well replicated, such as *ESR1*, *COL1A1*, *VDR*, *LRP5*, *OPG*, and *CYP19A1*.(8–16) Recent advances in single-nucleotide polymorphism (SNP) genotyping technologies and analytical methods have provided new opportunities for researchers to launch powerful genome-wide association (GWA) studies to discover common variants for BMD that have yielded certain results.(13,14,17,18) However, the variants identified by the previous genetic studies could explain, in combination, only a very small fraction (<10%) of the BMD variation. This means that many additional genetic variants underlying BMD have to be uncovered. Therefore, we performed a GWA study to identify novel genetic variants that may influence FN BMD.

## Materials and Methods

### Subjects

This study was approved by the required institutional review board or research administration of the involved institutions. Signed informed-consent documents were obtained from all study participants before entering the study. The basic characteristics of the study sample sets are summarized in Table 1, with additional descriptions below.

**Table 1:** Summary Characteristics of the Study Subjects

	Discovery sample	Replication sample	Total sample
Number assessed for BMD	983	2557	3540
Gender (males/females)	488/495	1148/1409	1636/1904
Age (years)	50.3 (18.3)	66.4 (11.6)	62.0 (15.6)
Weight (kg)	80.1 (17.7)	76.4 (17.3)	77.4 (17.5)
Height (cm)	170.8 (9.7)	165.5 (10.2)	167.0 (11.1)
Femoral neck BMD (g/cm^2^)	0.81 (0.14)	0.87 (0.17)	0.86 (0.16)

*Note*: Data are shown as mean (SD).

#### Discovery Sample

The discovery sample set was identified from our established and expanding database currently containing more than 10,000 subjects. This sample consisted of 983 unrelated healthy subjects (495 women and 488 men) who had both the phenotype and genotype information. All the subjects were white US citizens of northern European origin living in Omaha, Nebraska, and its surrounding regions in the Midwest. Subjects with chronic diseases and conditions that potentially might affect bone mass, structure, or metabolism were excluded from the study to minimize the influence of known environmental and therapeutic factors on bone variation. The exclusion criteria have been detailed in an earlier publication.(19) BMD measurements were obtained using dual-energy X-ray absorptiometry (DXA; Hologic QDR4500, Hologic Inc., Waltham, MA, USA) at the FN. The coefficient of variation (CV) value of the FN BMD was approximately 1.40%.

#### Replication Sample

The replication sample was derived from the Framingham Heart Study (FHS) SNP Health Association Resource (SHARe) Project, for which genotyping was conducted in over 9300 phenotyped subjects from three generations (including over 900 families). Details about and descriptions of the FHS were reported previously.(20,21) We have the data on 2557 phenotyped white subjects from 750 families. In this group, 841 subjects (325 men and 516 women) were from the original cohort, and 1716 (823 men and 893 women) were from the offspring cohort. The original cohort participants had BMD measures by DXA machine (Lunar DPX-L, Lunar Corp., Madison, WI, USA) at the FN performed at examination number 22. The offspring cohort participants were scanned with the same machine at examination 6 or 7. As reported previously,(21) the CV was 1.7% for FN.

### Genotyping and quality control

For the discovery sample, genomic DNA was extracted from whole human blood using a commercial isolation kit (Gentra Systems, Minneapolis, MN, USA) following the standard protocol. Genotyping was carried out at Vanderbilt Microarray Shared Resource using the Affymetrix Human Mapping 500K array set (Affymetrix, Santa Clara, CA, USA), as described in a previous publication.(22) The final average Bayesian Robust Linear Model with Mahalanobis distance classifier (BRLMM)(23) call rate across the entire sample reached a high level of 99.14%. However, of the initial full set of 500,568 SNPs, we discarded 32,961 SNPs with call rate of less than 95%, another 33,358 SNPs deviating from Hardy-Weinberg equilibrium (HWE; *p* < .0001), and 91,395 SNPs with minor allele frequencies (MAFs) of less than 5%. Therefore, the final analyses were restricted to 342,854 SNPs.

For the replication sample, genotyping was performed using approximately 550,000 SNPs (Affymetrix 500K mapping array plus Affymetrix 50K supplemental array). For details of the genotyping method, please refer to the FHS SHARe at the NCBI dbGaP Web site (http://www.ncbi.nlm.nih.gov/projects/gap/cgibin/study.cgi?study_id=phs000007.v3.p2). The quality control was the same as that adopted for the discovery sample by excluding SNPs with a call rate of less than 95%, deviating from HWE (*p* < .0001), and with MAFs of less than 5%. There were 386,731 SNPs available for subsequent analyses.

### Statistical analysis

The raw BMD values were adjusted by the significant covariates, including age, sex, and weight. The BMD residuals were used for subsequent association analyses. For the discovery sample, EIGENSTRAT(24) was applied to test for SNP associations, assuming an additive inheritance model. The first 10 principal components were selected to perform such analyses. For the follow-up replication sample, we selected the most significantly associated SNPs that reached a *p* < 5 × 10^−4^ (175 SNPs) to test for associations with FN BMD. FBAT(25) was used to examine family-based associations under the additive model.

Meta-analysis statistics were generated using the weighted *Z*-scores (a standard normal deviate, the statistic associated with a *p* value) to quantify the overall evidence for association with BMD. The individual *Z*-score was weighted by the square root of the sample size of each study. We added the individual weighted *Z*-scores derived from each sample together and divided by the square root of the sum of the sample sizes to obtain an overall *Z*-score and an associated combined *p* value.(26)

Combining results from all sample sets by meta-analysis, we set the threshold for genome-wide significance at *p* < 4.2 × 10^−7^ according to Freimer and Sabatti(27) and Lencz and colleagues,(28) who preferred a more accurate estimate by considering the total number of genes in the human genome. Moreover, a nominally significant association threshold (*p* < .05) was set in the replication stage to ensure that the overall significant association is robust across populations.

Haploview Version 4.1(29) was used to characterize linkage disequilibrium (LD, *r*^2^) pattern and plot the haplotype block patterns.

Different genotyping platforms were used in our GWA study and in previous BMD GWA studies.(13,14,17) For those reported promising SNPs that were missing in our Affymetrix 500K arrays, we imputed the genotypes using the IMPUTE program(30) in order to facilitate comparison of associations at the same SNPs. To ensure the reliability of the imputation, all the imputed SNPs have reached a calling threshold of 0.90, i.e., a 90% probability that an imputed genotype is true. SNPTEST(30) was used to test for associations between the imputed SNPs and FN BMD using age, sex, and weight as covariates.

## Results

We first carried out a GWA scan in the discovery sample of 983 unrelated white persons and then selected the top 175 most significantly associated SNPs with *p* < 5 × 10^−4^ (Supplemental Table 1) to test for associations in the replication sample of 2557 white persons from 750 families. Combining results from these two sample sets, we identified two promising loci, 11p15 and 16p11, that harbored a cluster of 6 SNPs ranked as the most significant SNPs among the list (Table 2). 11p15 was represented by three significant SNPs, which were rs9630182 (combined *p* = 3.98 × 10^−7^), rs2036417 (combined *p* = 9.52 × 10^−6^), and rs7125774 (combined *p* = 1.05 × 10^−5^), respectively. In particular, rs9630182 achieved the genome-wide significance level (*p* < 4.2 × 10^−7^). These three SNPs are highly correlated with one another (pairwise LD *r*^2^ > 0.99; 1*A*) and are located approximately 100 kb upstream of the parathyroid hormone (*PTH*) gene. The whole *PTH* gene, including these three SNPs (from upstream to downstream), was localized to a single block with a size of 125 kb (1*A*). This gene has been reported previously to be a potent modulator to regulate osteoblasts and to increase bone formation.(31,32) This is consistent with our findings that these three SNPs have a consistently protective effect on BMD because each copy of the minor allele of each SNP was associated with an increase in FN BMD by approximately 0.11 SD, as estimated in the discovery sample. The effect of each SNP in the replication sample was in the same direction as in the discovery sample. The variance in BMD variation explained by these three SNPs was 1.64% (rs9630182), 1.52% (rs2036417), and 1.38% (rs7125774), respectively. We also compared the distribution differences of genotype frequencies for the identified SNPs between the two studied samples and found no significant differences (*p* > .05) (Supplemental Table 2).

**Table 2:** Associations Between SNPs at the Two Promising Regions for BMD at the Femoral Neck

			Discovery sample			Replication sample		
					
SNP	Position	Alleles^a^	MAF	*p* Value	Effect size (SD)^b^	MAF	*p* Value	Combined *p* value
11p15 (*PTH*)								
rs9630182	13576748	T/C	0.345	1.10 × 10^−4^	0.1104	0.383	6.50 × 10^−4^	3.98 × 10^−7^
rs2036417	13574184	A/G	0.364	3.24 × 10^−4^	0.1101	0.386	5.08 × 10^−3^	9.52 × 10^−6^
rs7125774	13575380	C/T	0.357	3.06 × 10^−4^	0.1100	0.381	5.68 × 10^−3^	1.05 × 10^−5^
16p11 (*IL21R*)								
rs8057551	27342428	G/A	0.325	1.51 × 10^−4^	0.1102	0.317	2.36 × 10^−3^	2.31 × 10^−6^
rs8061992	27342539	A/C	0.335	1.53 × 10^−4^	0.1101	0.312	6.74 × 10^−3^	8.62 × 10^−6^
rs7199138	27342034	C/G	0.335	3.88 × 10^−4^	0.1103	0.315	6.41 × 10^−3^	1.41 × 10^−5^

The former allele represents the minor allele.

Effect size is the additive effect of each minor allele on the residual of femoral neck BMD (after adjustment for age, sex, and weight).

**Fig. 1:**
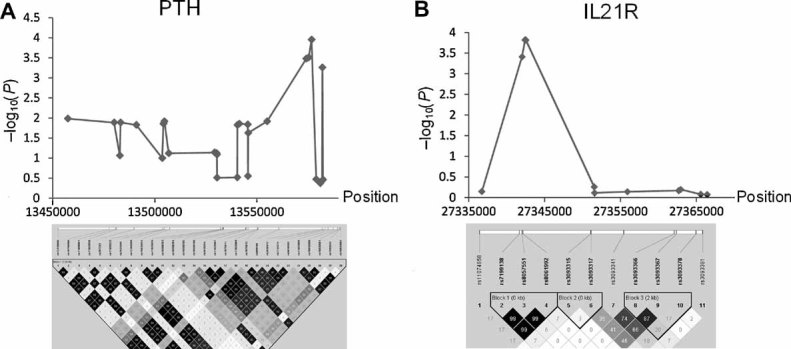
Pair-wise linkage disequilibrium diagrams for two promising loci: (*A*) *PTH*; (*B*) *IL21R*. Pair-wise linkage disequilibrium (LD), measured as *r*^2^, was calculated from genotyping data in the discovery sample using the HAPLOVIEW program. Shading represents the magnitude of pair-wise LD, with a white-to-black gradient reflecting lower to higher LD values. The scatter graph indicates the negative logarithm of *p* value for each SNP in the discovery sample. The *x* axis denotes the genomic position.

Another promising loci, 16p11, contains three significant SNPs, rs8057551 (combined *p* = 2.31 × 10^−6^), rs8061992 (combined *p* = 8.62 × 10^−6^), and rs7199138 (combined *p* = 1.41 × 10^−5^). Although these three SNPs did not reach genome-wide significance, they are clustered in a potential candidate gene, interleukin 21 receptor (*IL21R*). This gene is a cytokine receptor that is important to bone biology. The three SNPs are in strong LD with each other (*r*^2^ > 0.95) and are located in an LD block within intron 1 of *IL21R* (Fig. 1B). All three of these SNPs were associated with an increased FN BMD value in both the discovery and the replication samples, with the effect size estimated to be approximately 0.11 SD for each minor allele of each SNP in the discovery sample. The contribution of the three SNPs to BMD variation was 1.47% (rs8057551), 1.40% (rs8061992), and 1.22% (rs7199138), respectively.

We further performed gender-specific association analyses (adjusted for age and weight) for the preceding 6 SNPs in *PTH* and *IL21R*. The significant associations in the total sample could be generally replicated in each gender group (Table 3). For *PTH*, the associations were driven mainly by male subjects, whereas for *IL21R*, the associations were caused mainly by female subjects, as reflected in the discovery sample. Overall, the association signals in each gender group generally were weaker than in the total sample, which might be largely due to the smaller sample sizes in each gender group.

**Table 3:** Gender-Specific Association Signals for the Six SNPs Identified for BMD at the Femoral Neck

	Discovery sample *p* value		Replication sample *p* value	
		
SNP	Male	Female	Male	Female
11p15 (*PTH*)				
rs9630182	3.56 × 10^−4^	0.051	5.11 × 10^−3^	0.022
rs2036417	3.88 × 10^−4^	0.121	0.026	0.062
rs7125774	5.06 × 10^−4^	0.059	0.032	0.046
16p11 (*IL21R*)				
rs8057551	0.010	5.60 × 10^−3^	0.020	0.034
rs8061992	0.011	6.37 × 10^−3^	0.034	0.055
rs7199138	0.021	9.35 × 10^−3^	0.032	0.048

Using the genotyped and imputed genotypes in our GWA discovery sample of 983 unrelated white persons, we examined the associations between FN BMD and the key SNPs identified in previous GWA studies.(13,14,17) Five SNPs were confirmed to be associated with FN BMD in our sample, including rs851982 (*p* = .012) and rs4870044 (*p* = .045) in *ESR1*, rs6469804 (*p* = .030) in *OPG*, rs3736228 (*p* = .048) in *LRP5*, and rs2010281 (*p* = .048) in *MARK3* (Table 4). Moreover, another two new SNPs in *LRP5* also were found to be associated with FN BMD in our sample (i.e., rs604944, *p* = 5.3 × 10^−4^, and rs4988327, *p* = 3.6 × 10^−3^). Meanwhile, for SNPs that were not confirmed in our sample, we list the results in Supplemental Table 3 for reference.

**Table 4:** Comparison of the Previous GWA Studies for BMD and the Current GWA Study

SNP	Associated gene	Cytoband	Current GWA *p* value	Published GWA *p* value^a^	Reference
rs851982	*ESR1*	6q25	0.012	1.6 × 10^−5^ (hip BMD^b^)	14
rs4870044	*ESR1*	6q25	0.045	9.9 × 10^−5^ (hip BMD)	14
rs6469804	*OPG*	8q24	0.030	1.6 × 10^−4^ (SPBMD^c^)	13
				0.04 (hip BMD)	14
rs3736228	*LRP5*	11q13	0.048	1.9 × 10^−5^ (SPBMD)	13
rs604944	*LRP5*	11q13	5.3 × 10^−4^	—	
rs4988327	*LRP5*	11q13	3.6 × 10^−3^	—	
rs2010281	*MARK3*	14q32	0.023	7.4 × 10^−5^ (hip BMD)	17

a *p* value reported here was the original *P* value in the discovery sample in each GWA study.

b Hip BMD is the combined BMD at the femoral neck, trochanter, and intertrochanter region.

c SPBMD = spine BMD.

## Discussion

The GWA approach is a state-of-the-art approach to uncover modest genetic variants contributing to common diseases or phenotypes. Using a GWA approach, our group has reported two candidate genes—*ADAMTS18* (16q23) and *TGFBR3* (1p22)—for spine or hip BMD previously.(18) In addition, three other GWA studies on BMD have been published,(13,14,17) and they successfully identified several candidate genes for BMD, including *RANKL* (13q14), *OPG* (8q24), *RANK* (18q21), *ESR1* (6q25), *LRP5* (11q13), *SOST* (17q21), *MARK3* (14q32), and *SP7* (12q13). However, these loci in combination can explain only a small fraction of BMD variation, leaving the majority of the genetic factors that influence BMD variation unknown. In addition, most published GWA studies focused only on the genes or SNPs of top-ranking statistical significance, which may ignore some useful information. In this study, by using available GWA data sets from two white populations, we identified two susceptibility genes—*PTH* (11p15) and *IL21R* (16p11)—associated with FN BMD variation. These two genes were not in the top-significance list in either of the populations and were not identified by our previous GWA study on BMD.(18) However, combining the two data sets by meta-analysis revealed the promising significance of these two genes because the meta-analysis could improve the power to detect more associations and investigate the consistency of those associations across different populations.(33) Moreover, both genes have potential biologic functions that are important to bone metabolism. Thus our findings added more information to the overall understanding of the genetic basis of osteoporosis.

*PTH* plays a pivotal role in calcium homeostasis and bone remodeling. In experimental animals and patients with osteoporosis, intermittent administration of PTH can increase bone mass by stimulating de novo bone formation.(32,34–36) However, genetic studies testing for association between polymorphisms in *PTH* and osteoporosis are lacking, and most of them are underpowered and show inconsistent results.(37–41) Our study found a consistent association between *PTH* and FN BMD in two independent white populations, thereby supporting the conclusion that *PTH* is an important candidate gene for BMD and osteoporosis. Although the significant SNPs we identified are located in the upstream of the *PTH* gene, they are clustered in the same LD block as the SNPs within the *PTH* gene. In addition, intergenic transcription now has been recognized as an active and common cellular process. Extensive transcription has been observed in unannotated genomic regions that are related to genotype-phenotype correlations.(42,43) As an important function, intergenic transcription can regulate expression of the nearby genes.(44,45) In particular, SNPs rs9630182 and rs2036417 are located at potential transcription factor binding sites predicted by the FASTSNP program (http://fastsnp.ibms.sinica.edu.tw). Thus we hypothesized that those SNPs potentially might regulate *PTH* gene expression through intergenic transcription, although the real molecular mechanisms await further investigation.

Cytokins are potent mediators regulating homeostasis of the immune system and pathophysiologic processes. As a member of the type I cytokine receptors, *IL21R* has multiple functions. For example, *IL21R* plays an important role in the proliferation and differentiation of various immune cells, such as T cells and B cells. Studies have shown that B cells may participate in osteoclastogenesis.(46) *IL21R* induces the growth-promoting signals of its ligand, *IL21*, which might be involved in the maturation and function of myeloid cells.(47) *IL21R* and *IL21* have been revealed to be involved in a variety of human diseases, including cancers, inflammatory bowel disease and Crohn's disease, and multiple autoimmune diseases. Especially, *IL21R* has been identified as associated with the activated phenotype of rheumatoid arthritis fibroblasts and correlates negatively with the destruction of cartilage and bone.(48) With this information taken together, we suggested that *IL21R* may be a new candidate gene for BMD.

We compared the results for the key SNPs identified in previous BMD GWA studies(13,14,17) with our current GWA study. Since replication analysis was the specific hypothesis driven, *p* < .05 was considered significant. We confirmed associations for several SNPs located in the previously well-studied candidate genes, such as *ESR1*, *OPG*, and *LRP5* (Table 4). However, some SNPs were not able to be replicated in our study (Supplemental Table 3), which might be affected by many factors. First, the effect sizes of variants were very small and thus easily lead to failure of replication under current statistical power. Second, some SNPs identified in previous studies were for spine BMD, and our study focused only on FN BMD. BMDs at different skeletal sites may have different genetic mechanisms. Third, the differences in gene-gene and gene-environment interactions between the two study sets may result in inconsistency in replication. In addition, other factors, such as differential LD and allele frequencies across populations, also may significantly influence the chance of replicating GWA results.

It is worth emphasizing that population stratification is unlikely to be a major concern in this GWA study. This is so first because we used EIGENSTRAT to perform GWA analyses in the discovery sample, which can control for potential population stratification effectively. Second, we used a family-based sample to perform replication analyses. Family-based samples are ideal for the follow-up validation of initial GWA findings(49) because they are robust to population stratification and essentially can eliminate the possible impact of population stratification. Thus our GWA results are not likely to be plagued by spurious associations owing to population stratification.

In summary, we identified two susceptibility bone mass candidate genes, *PTH* and *IL21R*, that may influence FN BMD variation. Although additional functional studies are required to elucidate the detailed roles and potential functional variants of these loci, our findings provide some new insights into the understanding of the genetic architecture of BMD and osteoporosis.
