# Supplementary material for: IL21R and PTH May Underlie Variation of Femoral Neck Bone Mineral Density as Revealed by a Genome-wide Association Study
Source: J Bone Miner Res. 2009 Oct 26;25(5):1042–8. doi: 10.1359/jbmr.091040 (PMC3153368; doi:10.1359/jbmr.091040)
Supplement: Supplementary file 1 [file jbmr0025-1042-SD1.doc]

Supplementary Table 1. Top 175 associated SNPs in the genome-wide scan

| SNP | Nearest Gene | Chromosome  Position | Allele | MAF | *P* value  discovery sample | *P* value  replication sample | Combined *P* value |
| --- | --- | --- | --- | --- | --- | --- | --- |
| rs11015150 | APBB1IP | chr10_26852317 | A/G | 0.17 | 6.80×10^-6^ | 0.68 | 3.24×10^-3^ |
| rs17237198 |  | chr12_47240199 | A/G | 0.08 | 7.24×10^-6^ | 0.89 | 6.53×10^-3^ |
| rs2705141 | OR8S1 | chr12_47210694 | A/G | 0.08 | 1.07×10^-5^ | 0.94 | 8.56×10^-3^ |
| rs2705155 | C12orf54 | chr12_47193357 | C/T | 0.10 | 1.11×10^-5^ | 0.69 | 3.96×10^-3^ |
| rs3739461 | RFX3 | chr9_3385848 | C/T | 0.22 | 1.15×10^-5^ | 0.22 | 3.98×10^-4^ |
| rs7681750 |  | chr4_ 160442008 | A/G | 0.19 | 1.18×10^-5^ | 0.39 | 1.18×10^-3^ |
| rs9624295 | MIF | chr22_ 22374488 | C/G | 0.40 | 1.71×10^-5^ | 0.93 | 9.63×10^-3^ |
| rs17502069 | L3MBTL4 | chr18_ 6318558 | A/T | 0.13 | 2.17×10^-5^ | 0.53 | 2.78×10^-3^ |
| rs2081748 |  | chr12_ 113780260 | C/G | 0.05 | 2.37×10^-5^ | NA | NA |
| rs2731071 | OR8S1 | chr12_47203274 | C/T | 0.10 | 3.79×10^-5^ | 0.68 | 5.84×10^-3^ |
| rs10399860 | CHRM3 | chr1_ 236390579 | A/G | 0.29 | 4.45×10^-5^ | 0.28 | 1.07×10^-3^ |
| rs3898848 | L3MBTL4 | chr18_ 6284119 | C/T | 0.18 | 4.46×10^-5^ | 0.11 | 2.24×10^-4^ |
| rs532419 | NGFB | chr1_ 115788208 | A/T | 0.31 | 4.82×10^-5^ | 0.74 | 7.68×10^-3^ |
| rs591004 | LAMA1 | chr18_7012495 | C/T | 0.30 | 5.16×10^-5^ | 0.10 | 2.06×10^-4^ |
| rs158690 | SYK | chr9_90736540 | A/G | 0.12 | 5.81×10^-5^ | 0.07 | 1.26×10^-4^ |
| rs2249959 | SIM2 | chr21_ 37019091 | A/G | 0.31 | 5.82×10^-5^ | 0.58 | 4.81×10^-3^ |
| rs17395860 | SLC10A2 | chr13_ 103151946 | C/G | 0.14 | 6.20×10^-5^ | 0.3 | 1.38×10^-3^ |
| rs1347677 | SOX6 | chr11_16252988 | G/T | 0.20 | 6.25×10^-5^ | NA | NA |
| rs10035791 | HINT1 | chr5_129919660 | A/G | 0.17 | 7.32×10^-5^ | 0.95 | 1.60×10^-2^ |
| rs17744331 | TRHDE | chr12_ 71629596 | A/T | 0.22 | 7.37×10^-5^ | 0.72 | 8.34×10^-3^ |
| rs2219866 |  | chr3_151243953 | C/T | 0.06 | 7.44×10^-5^ | 0.81 | 1.09×10^-2^ |
| rs1627493 | P15RS | chr18_31832437 | A/T | 0.18 | 7.62×10^-5^ | 0.67 | 7.20×10^-3^ |
| rs184649 | L3MBTL4 | chr10_109949274 | A/G | 0.18 | 8.04×10^-5^ | 0.07 | 1.48×10^-4^ |
| rs2484591 |  | chr12_113789177 | A/G | 0.06 | 8.06×10^-5^ | NA | NA |
| rs1896331 |  | chr12_113789692 | C/G | 0.06 | 8.15×10^-5^ | NA | NA |
| rs13076437 | GRM7 | chr3_6088535 | A/C | 0.28 | 8.27×10^-5^ | 0.43 | 3.02×10^-3^ |
| rs10515709 |  | chr5_154953129 | A/G | 0.22 | 8.85×10^-5^ | 0.69 | 8.09×10^-3^ |
| rs10904976 | PRKCQ | chr10_6877791 | A/G | 0.17 | 1.05×10^-4^ | 0.14 | 4.86×10^-4^ |
| rs576550 | ATPIF1 | chr1_28250750 | A/C | 0.28 | 1.06×10^-4^ | 0.44 | 3.47×10^-3^ |
| rs10958891 |  | chr9_10114871 | G/T | 0.11 | 1.06×10^-4^ | 0.84 | 1.34×10^-2^ |
| rs10832032 | BTBD10 | chr11_13367920 | A/C | 0.44 | 1.06×10^-4^ | 0.04 | 7.61×10^-5^ |
| rs360074 | LEFTY1 | chr1_222397885 | A/G | 0.45 | 1.07×10^-4^ | 0.31 | 1.84×10^-3^ |
| rs11022781 | ARNTL | chr11_13358985 | C/T | 0.45 | 1.08×10^-4^ | 0.05 | 1.05×10^-4^ |
| rs1552013 | C17orf54 | chr17_69248671 | C/T | 0.11 | 1.08×10^-4^ | 0.21 | 9.50×10^-4^ |
| **rs9630182** | **PTH** | **chr11_13576748** | **C/T** | **0.34** | **1.10×10^-4^** | **6.50×10^-4^** | **3.98×10^-7^** |
| rs17127848 | DLC1 | chr8_ 13159076 | A/G | 0.08 | 1.12×10^-4^ | 0.34 | 2.21×10^-3^ |
| rs12795885 | TMEM16C | chr11_26618623 | C/T | 0.45 | 1.15×10^-4^ | 0.60 | 6.61×10^-3^ |
| rs186884 | L3MBTL4 | chr18_ 6327489 | A/C | 0.18 | 1.18×10^-4^ | 0.12 | 4.05×10^-4^ |
| rs7235294 | L3MBTL4 | chr18_6300558 | C/T | 0.18 | 1.19×10^-4^ | 0.15 | 5.74×10^-4^ |
| rs2601552 | L3MBTL4 | chr18_6340286 | A/G | 0.18 | 1.20×10^-4^ | 0.07 | 1.81×10^-4^ |
| rs1928295 | TRIM32 | chr9_117458037 | A/G | 0.44 | 1.21×10^-4^ | 0.67 | 8.47×10^-3^ |
| rs41406246 | LEF1 | chr4_109559025 | C/T | 0.16 | 1.21×10^-4^ | 0.03 | 5.45×10^-5^ |
| rs10953363 | PRKRIP1 | chr7_101617965 | A/T | 0.48 | 1.22×10^-4^ | 0.91 | 1.69×10^-2^ |
| rs648210 |  | chr10_20589237 | A/G | 0.42 | 1.24×10^-4^ | 0.87 | 1.53×10^-2^ |
| rs10835040 | TMEM16C | chr11_26619970 | A/G | 0.45 | 1.24×10^-4^ | 0.64 | 7.76×10^-3^ |
| rs2771335 | METT11D1 | chr14_20523213 | C/G | 0.40 | 1.28×10^-4^ | 0.17 | 7.25×10^-4^ |
| rs9903519 | SDK2 | chr17_69248565 | C/T | 0.11 | 1.29×10^-4^ | 0.15 | 5.95×10^-4^ |
| rs9909176 | C17orf54 | chr17_ 69248438 | C/T | 0.11 | 1.30×10^-4^ | 0.18 | 8.01×10^-4^ |
| rs4489787 | ANP32D | chr12_47097367 | C/T | 0.11 | 1.34×10^-4^ | 0.72 | 1.02×10^-2^ |
| rs9408902 | ASTN2 | chr9_117458518 | A/G | 0.44 | 1.35×10^-4^ | 0.55 | 5.87×10^-3^ |
| rs908606 | GRM7 | chr3_6095251 | C/G | 0.37 | 1.39×10^-4^ | 0.06 | 1.55×10^-4^ |
| rs307246 | BTBD10 | chr11_13439803 | A/G | 0.44 | 1.40×10^-4^ | 0.03 | 5.88×10^-5^ |
| rs417757 | L3MBTL4 | chr18_6267575 | C/T | 0.18 | 1.42×10^-4^ | 0.06 | 1.57×10^-4^ |
| rs4908393 | DNAJC8 | chr1_ 28234590 | A/G | 0.28 | 1.43×10^-4^ | 0.65 | 8.44×10^-3^ |
| **rs8057551** | **IL21R** | **chr16_27342428** | **A/G** | **0.32** | **1.51×10^-4^** | **2.36×10^-3^** | **2.31×10^-6^** |
| **rs8061992** | **IL21R** | **chr16_27342539** | **A/C** | **0.33** | **1.53×10^-4^** | **6.74×10^-3^** | **8.62×10^-6^** |
| rs9508 | ATPIF1 | chr1_28436866 | C/T | 0.28 | 1.53×10^-4^ | 0.56 | 6.38×10^-3^ |
| rs7008117 | ATP6V1H | chr8_ 54747208 | G/T | 0.23 | 1.62×10^-4^ | 0.10 | 3.55×10^-4^ |
| rs2609495 | CACNA1E | chr1_177983271 | A/G | 0.25 | 1.63×10^-4^ | 0.54 | 6.08×10^-3^ |
| rs17369045 | PDE3A | chr12_ 20022230 | C/T | 0.06 | 1.67×10^-4^ | 0.06 | 1.70×10^-4^ |
| rs1527781 |  | chr11_ 131208234 | A/G | 0.25 | 1.71×10^-4^ | 0.28 | 1.87×10^-3^ |
| rs7777879 | RPA3 | chr7_ 7482577 | A/T | 0.10 | 1.73×10^-4^ | 0.18 | 9.10×10^-4^ |
| rs11222814 |  | chr11_ 131215220 | A/G | 0.44 | 1.74×10^-4^ | 0.35 | 2.77×10^-3^ |
| rs290221 | SYK | chr9_ 90722321 | G/T | 0.10 | 1.75×10^-4^ | 0.08 | 2.64×10^-4^ |
| rs4242909 |  | chr12_ 131789451 | A/G | 0.43 | 1.78×10^-4^ | 0.39 | 3.41×10^-3^ |
| rs10894455 |  | chr11_ 131201194 | C/T | 0.24 | 1.85×10^-4^ | 0.48 | 3.27×10^-3^ |
| rs6679677 | PTPN22 | chr1_ 114015850 | A/C | 0.10 | 1.87×10^-4^ | 0.66 | 9.58×10^-3^ |
| rs13192934 | TCBA1 | chr6_124286263 | G/T | 0.31 | 1.89×10^-4^ | 0.90 | 1.90×10^-2^ |
| rs6484237 | TMEM16C | chr11_ 26631344 | C/T | 0.46 | 1.92×10^-4^ | 0.82 | 1.54×10^-2^ |
| rs17833456 | TNP1 | chr2_ 217688387 | A/G | 0.34 | 1.95×10^-4^ | 0.93 | 2.08×10^-2^ |
| rs10755296 |  | chr5_73016625 | C/G | 0.29 | 1.96×10^-4^ | 0.73 | 1.20×10^-2^ |
| rs1981810 |  | chr5_72955334 | A/G | 0.27 | 1.97×10^-4^ | 0.68 | 1.03×10^-2^ |
| rs684634 | LAMA1 | chr18_ 7013371 | A/G | 0.36 | 2.04×10^-4^ | 0.16 | 8.13×10^-4^ |
| rs4497357 | TMEM16C | chr11_ 26619122 | C/T | 0.45 | 2.06×10^-4^ | 0.35 | 2.98×10^-3^ |
| rs16862394 |  | chr3_ 151237654 | C/T | 0.07 | 2.07×10^-4^ | 0.73 | 1.22×10^-2^ |
| rs1452539 | L3MBTL4 | chr18_6277726 | A/G | 0.18 | 2.09×10^-4^ | 0.05 | 1.47×10^-4^ |
| rs9289800 |  | chr3_ 151243166 | A/G | 0.07 | 2.11×10^-4^ | 0.75 | 1.31×10^-2^ |
| rs2612543 | SETBP1 | chr18_ 41270199 | G/T | 0.17 | 2.15×10^-4^ | 0.15 | 7.52×10^-4^ |
| rs427574 | L3MBTL4 | chr18_6306237 | C/G | 0.18 | 2.19×10^-4^ | 0.05 | 1.51×10^-4^ |
| rs12197564 |  | chr6_ 45900148 | G/T | 0.35 | 2.22×10^-4^ | 0.09 | 3.53×10^-4^ |
| rs529566 |  | chr11_ 131200026 | A/G | 0.23 | 2.25×10^-4^ | 0.54 | 6.86×10^-3^ |
| rs7217284 | BCAS3 | chr17_ 56691176 | G/T | 0.07 | 2.32×10^-4^ | 0.46 | 5.11×10^-3^ |
| rs10505844 | PDE3A | chr12_ 20016609 | C/G | 0.06 | 2.34×10^-4^ | 0.10 | 4.24×10^-4^ |
| rs6438523 | CDGAP | chr3_ 120527813 | A/G | 0.40 | 2.44×10^-4^ | 0.45 | 5.00×10^-3^ |
| rs6874868 | HINT1 | chr5_ 129825862 | A/G | 0.17 | 2.50×10^-4^ | 0.38 | 3.72×10^-3^ |
| rs10832036 | BTBD10 | chr11_13419429 | G/T | 0.44 | 2.52×10^-4^ | 0.03 | 8.07×10^-5^ |
| rs275344 | PLEKHG1 | chr6_ 151053202 | C/T | 0.26 | 2.57×10^-4^ | 0.79 | 1.56×10^-2^ |
| rs2013565 | TSSC1 | chr2_ 4671884 | C/T | 0.07 | 2.57×10^-4^ | 0.35 | 3.52×10^-3^ |
| rs653152 | CLEC4M | chr19_ 7746343 | A/G | 0.29 | 2.58×10^-4^ | 0.10 | 4.44×10^-4^ |
| rs2543047 |  | chr8_ 39938823 | A/G | 0.37 | 2.59×10^-4^ | NA | NA |
| rs9400929 |  | chr6_116936016 | A/G | 0.44 | 2.59×10^-4^ | 0.84 | 1.80×10^-2^ |
| rs11179448 | TRHDE | chr12_ 71567861 | A/G | 0.18 | 2.62×10^-4^ | 0.93 | 2.28×10^-2^ |
| rs16953178 | ERCC4 | chr16_ 13617638 | C/T | 0.05 | 2.65×10^-4^ | 0.61 | 9.25×10^-3^ |
| rs4835994 | CHSY-2 | chr5_ 129749756 | A/G | 0.18 | 2.74×10^-4^ | 0.64 | 1.03×10^-2^ |
| rs11634290 | CSPG4 | chr15_ 73771150 | C/T | 0.48 | 2.75×10^-4^ | 0.34 | 3.18×10^-3^ |
| rs12602529 | C17orf54 | chr17_ 69246972 | C/T | 0.10 | 2.76×10^-4^ | 0.11 | 5.29×10^-4^ |
| rs17368064 | PDE3A | chr12_ 19987293 | A/G | 0.05 | 2.86×10^-4^ | 0.07 | 2.78×10^-4^ |
| rs7769605 | PLEKHG1 | chr6_ 151049983 | A/G | 0.26 | 2.90×10^-4^ | 0.84 | 1.87×10^-2^ |
| rs11720469 |  | chr3_ 167963910 | C/T | 0.41 | 2.90×10^-4^ | 0.57 | 8.37×10^-3^ |
| rs11147041 |  | chr12_ 131929010 | G/T | 0.32 | 2.93×10^-4^ | 0.34 | 3.27×10^-3^ |
| rs6786522 | CDGAP | chr3_ 120515050 | C/G | 0.41 | 2.95×10^-4^ | 0.44 | 5.18×10^-3^ |
| rs7744763 | TCBA1 | chr6_ 124287535 | A/G | 0.31 | 2.96×10^-4^ | 0.80 | 1.69×10^-2^ |
| rs1670131 | L3MBTL4 | chr18_ 6305744 | A/C | 0.17 | 3.02×10^-4^ | 0.24 | 1.84×10^-3^ |
| rs10958910 |  | chr9_ 10124760 | C/T | 0.11 | 3.04×10^-4^ | 0.97 | 2.65×10^-2^ |
| rs4551717 | TMEM16C | chr11_ 26619314 | A/G | 0.47 | 3.05×10^-4^ | 0.09 | 4.13×10^-4^ |
| **rs7125774** | **PTH** | **chr11_** **13575380** | **C/T** | **0.35** | **3.06×10^-4^** | **5.68×10^-3^** | **1.05×10^-5^** |
| rs17799756 | TRHDE | chr12_ 71580046 | A/G | 0.18 | 3.07×10^-4^ | 0.99 | 2.79×10^-2^ |
| rs17127858 | DLC1 | chr8_ 13159461 | A/C | 0.07 | 3.11×10^-4^ | 0.34 | 3.35×10^-3^ |
| rs991795 | TCBA1 | chr6_ 124287039 | C/G | 0.31 | 3.13×10^-4^ | 0.96 | 2.60×10^-2^ |
| rs17488764 |  | chr5_ 93661050 | A/G | 0.12 | 3.18×10^-4^ | 0.23 | 1.75×10^-3^ |
| rs10958907 |  | chr9_ 10121581 | C/G | 0.11 | 3.24×10^-4^ | 0.85 | 1.98×10^-2^ |
| **rs2036417** | **PTH** | **chr11_** **13574184** | **A/G** | **0.36** | **3.24×10^-4^** | **5.08×10^-3^** | **9.52×10^-6^** |
| rs16937372 | EYA1 | chr8_ 72178786 | C/T | 0.05 | 3.29×10^-4^ | 0.36 | 3.78×10^-3^ |
| rs1448434 | CDGAP | chr3_ 120525701 | C/G | 0.41 | 3.33×10^-4^ | 0.47 | 6.12×10^-3^ |
| rs7649930 | CDGAP | chr3_ 120517200 | A/C | 0.41 | 3.34×10^-4^ | 0.42 | 5.00×10^-3^ |
| rs7129266 |  | chr11_ 131219413 | C/G | 0.25 | 3.36×10^-4^ | 0.43 | 5.23×10^-3^ |
| rs882311 | ADARB2 | chr10_ 1219548 | C/T | 0.28 | 3.39×10^-4^ | 0.14 | 8.37×10^-4^ |
| rs2602027 | TTC23 | chr15_ 97521295 | C/T | 0.19 | 3.44×10^-4^ | 0.70 | 1.34×10^-2^ |
| rs7646718 |  | chr3_ 167964244 | A/C | 0.41 | 3.49×10^-4^ | 0.49 | 6.73×10^-3^ |
| rs1490359 | TCBA1 | chr6_ 124277920 | A/G | 0.32 | 3.50×10^-4^ | 0.95 | 2.63×10^-2^ |
| rs9318741 |  | chr13_ 80649409 | C/T | 0.06 | 3.61×10^-4^ | 0.65 | 1.17×10^-2^ |
| rs10039487 | HINT1 | chr5_ 129980823 | A/G | 0.18 | 3.61×10^-4^ | 0.80 | 1.81×10^-2^ |
| rs1424413 | GALNT1 | chr18_ 31579930 | A/G | 0.30 | 3.67×10^-4^ | 0.95 | 2.67×10^-2^ |
| rs703561 | PAH | chr9_ 2626161 | A/G | 0.19 | 3.69×10^-4^ | 0.45 | 5.89×10^-3^ |
| rs2890901 |  | chr9_ 10102766 | C/T | 0.12 | 3.76×10^-4^ | 0.75 | 1.59×10^-2^ |
| rs2007112 | DLC1 | chr8_13152374 | A/G | 0.24 | 3.81×10^-4^ | 0.28 | 2.63×10^-3^ |
| rs658209 | LAMA1 | chr18_ 7013733 | C/G | 0.36 | 3.83×10^-4^ | 0.19 | 1.41×10^-3^ |
| **rs7199138** | **IL21R** | **chr16_** **27342034** | **C/G** | **0.33** | **3.88×10^-4^** | **6.41×10^-3^** | **1.41×10^-5^** |
| rs1904091 |  | chr9_ 1715917 | A/C | 0.10 | 3.92×10^-4^ | 0.98 | 2.94×10^-2^ |
| rs5755694 | MAPK1 | chr22_ 20525084 | A/G | 0.46 | 3.95×10^-4^ | 0.37 | 4.28×10^-3^ |
| rs4937657 |  | chr11_ 131201359 | C/T | 0.24 | 4.02×10^-4^ | 0.51 | 7.66×10^-3^ |
| rs10958892 |  | chr9_ 10115118 | C/T | 0.11 | 4.02×10^-4^ | 0.92 | 2.55×10^-2^ |
| rs12233670 | TLR1 | chr4_ 38609782 | C/T | 0.23 | 4.05×10^-4^ | 0.76 | 1.68×10^-2^ |
| rs10053765 |  | chr5_ 99021831 | A/G | 0.38 | 4.12×10^-4^ | 0.23 | 1.97×10^-3^ |
| rs3739319 | INDO | chr8_ 39904478 | C/T | 0.37 | 4.12×10^-4^ | 0.28 | 2.72×10^-3^ |
| rs17086323 | TCBA1 | chr6_ 124278943 | A/T | 0.31 | 4.13×10^-4^ | 0.78 | 1.79×10^-2^ |
| rs9443479 | IRAK1BP1 | chr6_ 78583513 | A/G | 0.13 | 4.13×10^-4^ | 0.09 | 4.80×10^-4^ |
| rs2728619 | PDE3A | chr12_ 20049011 | A/G | 0.08 | 4.13×10^-4^ | 0.17 | 1.23×10^-3^ |
| rs10898982 | PGM2L1 | chr11_ 73770916 | A/G | 0.47 | 4.14×10^-4^ | 0.44 | 5.92×10^-3^ |
| rs10958909 |  | chr9_ 10124671 | A/C | 0.11 | 4.16×10^-4^ | NA | NA |
| rs2245781 | SULF1 | chr8_ 70573330 | A/G | 0.25 | 4.16×10^-4^ | 0.10 | 5.61×10^-4^ |
| rs10094093 |  | chr8_ 4493322 | A/G | 0.20 | 4.17×10^-4^ | 0.83 | 2.05×10^-2^ |
| rs10958890 |  | chr9_10114584 | A/G | 0.12 | 4.18×10^-4^ | 0.83 | 2.05×10^-2^ |
| rs2729459 |  | chr8_ 39939387 | A/G | 0.36 | 4.21×10^-4^ | 0.79 | 1.85×10^-2^ |
| rs7827872 | CSMD1 | chr8_3359557 | C/G | 0.06 | 4.22×10^-4^ | 0.99 | 3.08×10^-2^ |
| rs7775839 |  | chr6_116927839 | C/T | 0.44 | 4.23×10^-4^ | 0.89 | 2.41×10^-2^ |
| rs677872 |  | chr9_ 107994095 | C/T | 0.39 | 4.25×10^-4^ | 0.03 | 1.07×10^-4^ |
| rs7901816 | DOCK1 | chr10_ 129291896 | C/T | 0.26 | 4.28×10^-4^ | 0.68 | 1.36×10^-2^ |
| rs1483051 | IRAK1BP1 | chr6_ 78528802 | A/G | 0.13 | 4.29×10^-4^ | 0.30 | 3.10×10^-3^ |
| rs17727883 | NMU | chr4_ 56478327 | C/G | 0.13 | 4.32×10^-4^ | 0.18 | 1.37×10^-3^ |
| rs1656184 |  | chr4_37112836 | G/T | 0.15 | 4.33×10^-4^ | 0.69 | 1.41×10^-2^ |
| rs403814 | L3MBTL4 | chr18_ 6272593 | A/C | 0.18 | 4.36×10^-4^ | 0.04 | 1.59×10^-4^ |
| rs4567422 | CNTN5 | chr11_ 99114856 | C/G | 0.20 | 4.38×10^-4^ | 0.54 | 8.81×10^-3^ |
| rs1913205 | IRAK1BP1 | chr6_ 78577302 | C/T | 0.13 | 4.39×10^-4^ | 0.09 | 4.95×10^-4^ |
| rs5751704 | LOC51233 | chr22_ 22326529 | A/T | 0.48 | 4.41×10^-4^ | 0.43 | 5.82×10^-3^ |
| rs2930373 |  | chr8_ 3752396 | G/T | 0.14 | 4.42×10^-4^ | 0.79 | 1.88×10^-2^ |
| rs1322159 |  | chr9_ 10122696 | A/C | 0.11 | 4.43×10^-4^ | 0.60 | 1.08×10^-2^ |
| rs2330578 | SLC2A11 | chr22_ 22347529 | A/T | 0.48 | 4.43×10^-4^ | 0.51 | 7.95×10^-3^ |
| rs2729460 |  | chr8_ 39939776 | A/C | 0.36 | 4.45×10^-4^ | 0.60 | 1.08×10^-2^ |
| rs31623 | CAMK4 | chr5_ 96453454 | A/C | 0.16 | 4.49×10^-4^ | 0.51 | 7.99×10^-3^ |
| rs17136804 | RPA3 | chr7_7543326 | C/T | 0.10 | 4.52×10^-4^ | 0.07 | 3.51×10^-4^ |
| rs7847362 |  | chr9_ 81619285 | A/C | 0.12 | 4.54×10^-4^ | 0.11 | 6.73×10^-4^ |
| rs10490132 | LRP2 | chr2_ 169834468 | G/T | 0.49 | 4.58×10^-4^ | 0.15 | 1.07×10^-3^ |
| rs658188 | LAMA1 | chr18_ 7013748 | A/C | 0.36 | 4.63×10^-4^ | 0.18 | 1.42×10^-3^ |
| rs1837096 | SOX6 | chr11_ 16219450 | A/G | 0.20 | 4.67×10^-4^ | 0.16 | 1.19×10^-4^ |
| rs10499317 | COX19 | chr7_ 785344 | C/G | 0.27 | 4.75×10^-4^ | 0.49 | 7.59×10^-3^ |
| rs9657539 | MSRA | chr8_ 10293089 | A/G | 0.12 | 4.77×10^-4^ | 0.92 | 2.70×10^-2^ |
| rs1948608 | OR5K4 | chr3_ 99546260 | A/G | 0.09 | 4.78×10^-4^ | 0.46 | 6.78×10^-3^ |
| rs3734309 | FAM26E | chr6_ 116944003 | C/T | 0.44 | 4.79×10^-4^ | 0.85 | 2.26×10^-2^ |
| rs1483050 | IRAK1BP1 | chr6_78528639 | A/C | 0.13 | 4.81×10^-4^ | 0.13 | 8.84×10^-4^ |
| rs7032255 | ASTN2 | chr9_ 117479641 | C/T | 0.35 | 4.81×10^-4^ | 0.82 | 2.10×10^-2^ |
| rs10984568 | DBC1 | chr9_119340303 | C/T | 0.35 | 4.82×10^-4^ | 0.50 | 7.82×10^-3^ |
| rs1517380 | GRM7 | chr3_ 6087739 | A/G | 0.28 | 4.92×10^-4^ | 0.73 | 1.66×10^-2^ |
| rs6060692 | PHF20 | chr20_ 33981339 | A/T | 0.15 | 4.94×10^-4^ | 0.68 | 1.44×10^-2^ |
| rs9294087 | IRAK1BP1 | chr6_ 78581437 | A/C | 0.13 | 4.97×10^-4^ | 0.23 | 2.15×10^-3^ |

Note: The SNPs are listed as they ranked in the genome-wide association scan of femoral neck BMD in the discovery sample. The former allele listed in the table represents the minor allele, and the minor allele frequency (MAF) is given for the discovery sample. The SNPs with the most significant *P* values in the combined samples are indicated in bold. NA means the *P* value is unavailable because the corresponding SNP is excluded by the quality control.

Supplementary Table 2. Comparison of genotype frequencies differences for the six SNPs identified for BMD at femoral neck in the two studied samples

|  | rs9630182 | | | rs2036417 | | rs7125774 | | rs8057551 | | rs8061992 | | rs7199138 | |
| --- | --- | --- | --- | --- | --- | --- | --- | --- | --- | --- | --- | --- | --- |
|  | Sample 1^a^ | Sample 2^b^ | Sample 1 | | Sample 2 | Sample 1 | Sample 2 | Sample 1 | Sample 2 | Sample 1 | Sample 2 | Sample 1 | Sample 2 |
| AA (%) | 9.77 | 10.06 | 12.32 | | 13.79 | 11.66 | 13.34 | 9.79 | 8.92 | 10.91 | 10.20 | 10.94 | 10.42 |
| AB (%) | 49.95 | 49.01 | 48.50 | | 48.42 | 48.34 | 48.21 | 45.26 | 43.83 | 44.85 | 41.38 | 44.88 | 41.39 |
| BB (%) | 40.28 | 40.93 | 36.18 | | 37.79 | 40 | 38.45 | 44.95 | 47.25 | 44.24 | 48.42 | 44.18 | 48.19 |
| *P* value^c^ | 0.913 | | 0.641 | | | 0.551 | | 0.618 | | 0.236 | | 0.261 | |

^a^ Sample 1: Discovery sample

^b^ Sample 2: We selected 1,014 unrelated subjects consisting of the parents from each family to represent replication sample to compare the distribution differences of genotype frequencies.

^c^ *P* value was calculated by the Chi-square test.

Supplementary Table 3. The SNPs identified in previous GWA studies for BMD and not confirmed by the current GWA study

| SNP | Associated gene | Cytoband | Current GWA *P* value | Published GWA  *P* value^a^ | Reference |
| --- | --- | --- | --- | --- | --- |
| rs9479055 | ESR1 | 6q25 | 0.16 | 7.0×10^-4^(hipBMD^b^) | (14) |
| rs1038304 | ESR1 | 6q25 | 0.25 | 1.8×10^-5^(hipBMD) | (14) |
| rs6929137 | ESR1 | 6q25 | 0.09 | 1.4×10^-5^(hipBMD) | (14) |
| rs1999805 | ESR1 | 6q25 | 0.12 | 0.002(hipBMD) | (14) |
| rs2504063 | ESR1 | 6q25 | 0.20 | 5.7×10^-8^(SPBMD^c^) | (14) |
| rs4355801 | OPG | 8q24 | 0.07 | 7.9×10^-4^(SPBMD) | (13) |
| rs6993813 | OPG | 8q24 | 0.06 | 0.03(hipBMD) | (14) |
| rs9594759 | RANKL | 13q14 | 0.37 | 4.5×10^-4^ (hipBMD) | (14) |
| rs9594738 | RANKL | 13q14 | 0.41 | 2.7×10^-4^(hipBMD) | (14) |
| rs3018362 | RANK | 18q21 | 0.25 | 3.5×10^-5^(hipBMD) | (17) |
| rs1513670 | SOST | 17q21 | 0.42 | 6.0×10^-5^(hipBMD) | (17) |
| rs7220711 | SOST | 17q21 | 0.18 | 1.4×10^-4^ (hipBMD) | (17) |
| rs1107748 | SOST | 17q21 | 0.27 | 7.2×10^-5^(hipBMD) | (17) |

^a^ *P* value reported here was the original *P* value in the discovery sample in each GWA study.

^b^ hipBMD is the combined BMD at the femoral neck, trochanter and intertrochanter region.

^c^ SPBMD: Spine BMD.
